# Supplementary material for: Mismatch: a comparative study of vitamin D status in British-Bangladeshi migrants
Source: Evol Med Public Health. 2021 Jan 25;9(1):164–73. doi: 10.1093/emph/eoab001 (PMC7928960; doi:10.1093/emph/eoab001)
Supplement: eoab001_Supplementary_Data [file eoab001_supplementary_data.docx]

Table S1: Other characteristics of the cohort

**Table values indicate number (% of group)**

| Characteristic | White British  n=54 | Bangladeshi migrants  n=49 | Bangladeshi sedentees  n=45 | Total cohort  n=148 | p |
| --- | --- | --- | --- | --- | --- |
| **Religion^a^** |  |  |  |  |  |
| Muslim | n/a | 49 (100.0) | 27 (61.4) |  | **<0.001** |
| Hindu | n/a | 0 (0.0) | 16 (36.4) |  |  |
| Other | n/a | 0 (0.0) | 1 (2.3) |  |  |
|  |  |  |  |  |  |
| **Lifestyle** |  |  |  |  |  |
| Smoke at time of interview^b^ | 7 (13.7) | 0 (0.0) | 0 (0.0) | 7 (4.8) | **0.001** |
| Use betel nut^c^ | 0 (0.0) | 22 (45.8) | 25 (56.8) | 47 (32.2) | **<0.001** |
| Use betel nut with tobacco^d^ | 0 (0.0) | 15 (30.6) | 21 (48.8) | 36 (24.7) | **<0.001** |
| Take calcium supplements^e^ | 6 (11.3) | 3 (6.5) | 7 (15.6) | 16 (11.1) | 0.390 |
| Take 'other' supplements^f^ | 20 (37.0) | 14 (28.6) | 9 (20.5) | 43 (29.3) | 0.198 |

p-values in **bold** indicate significant difference between the groups (p<0.05).

^a^ Data for religion was not available for all White British women and n=1 sedentee.^b^ missing data for n=3 White British.^c^ missing data for n=1 migrant and n=1 sedentee

^d^ missing data for n=2 sedentees. ^e^ missing data for n=1 White British, n=3 migrants. ^f^ missing data for n=1 sedentee

Table S2: Season of serum sampling in the White British and Bangladeshi Migrant groups

|  | **‘Summer’ (May-Oct)** | **‘Winter’ (Nov-Apr)** | **Total** |
| --- | --- | --- | --- |
| **White British** | 38 (70.4%) | 16 (29.6%) | 54 |
| **Bangladeshi migrant** | 33 (67.3%) | 16 (32.7%) | 49 |
| **Total** | 71 (68.9%) | 32 (31.1%) | 103 |

Fisher’s exact test (2-sided) p=0.832

Table S3: Vitamin D insufficiency and low iron status

|  | **Ferritin:**  **Low (<13ng/ml)** | **Ferritin:**  **Adequate (13-150ng/ml)^a^** | **Total** |
| --- | --- | --- | --- |
| **Vitamin D: Insufficient(≤50nmol/L)** | 22  (25·6%) | 64  (74·4%) | 86  (100%) |
| **Vitamin D:**  **Sufficient (>50nmol/L)** | 6  (10·5%) | 51  (89·5%) | 57  (100%) |
| **Total** | 28  (19·6%) | 115  (80·4%) | 143  (100%) |

Fisher’s exact test (1-sided) p=0·02

^a^Five participants with abnormally high ferritin (>150ng/ml) were excluded from analysis (n=3 white, n=1 migrant, n=1 sedentee).

Women who were 25(OH)D insufficient (total n=89) had a significantly higher BMI than those who were ‘sufficient’ (n=59; mean BMI in insufficient group 26.6 vs 24.7 in sufficient group, p=0.009). When women were grouped by BMI into ‘healthy/underweight’ (<25) and ‘overweight/obese’ (≥25) the result was similar: mean 25(OH)D in the former category was 49.8nmol/L compared to 43.4nmol/L in the latter (p=0.043). Vitamin D levels were significantly negatively associated with increased tricep skin fold thickness and increased BMI but not with increased arm circumference. This remained true when these variables were analysed again, looking at mean values in women grouped into ‘insufficient’ (in vitamin D) vs ‘sufficient’ (Tables S3 and S4).

Table S4: Anthropometric measures and association with vitamin D as a continuous variable

| **Variable** | **Pearson correlation** | **p-value** |
| --- | --- | --- |
| BMI | -0.172 | **0.037** |
| WHR^a^ | -0.155 | 0.063 |
| Tricep skin fold thickness^b^ (cm) | -0.239 | **0.004** |
| Arm circumference^c^ (cm) | -0.116 | 0.169 |

Values in **bold** indicate significance (p=0.05)

^a^ missing data for n=3 whites, n=1 migrant

^b^ missing data for n=2 whites, n=1 migrant

^c^ missing data for n=5 whites, n=1 migrant

Table S5: Anthropometric measures and association with insufficiency vs sufficiency status

| **Variable** | **Mean in ‘insufficient’ women (<50nmol/L)**  **n=89** | **Mean in ‘sufficient’ women (≥50nmol/L)**  **n=59** | **p-value** |
| --- | --- | --- | --- |
| BMI | 26.6 | 24.7 | **0.009** |
| WHR^a^ | 0.828 | 0.812 | 0.177 |
| Tricep skin fold thickness^b^ (cm) | 25.9 | 21.7 | **0.002** |
| Arm circumference^c^ (cm) | 29.2 | 28.0 | 0.095 |

Values in **bold** indicate significance (p=0.05)

^a^ missing data for n=3 whites, n=1 migrant

^b^ missing data for n=2 whites, n=1 migrant

^c^ missing data for n=5 whites, n=1 migrant

## Other cohort characteristics and vitamin D

There were no significant associations between age and Vitamin D when analysing the entire sample, or within each group separately. Educational status (defined by number of years of education, grouped into <10 years or ≥10 years) appeared to be significantly associated with concentrations of 25(OH)D (mean in <10 years education=42.8nmol/L, ≥10 years=50.5nmol/L, p=0.017). However, as there were many more migrants compared to whites in the <10 years education group, linear regression also adjusting for group was performed. This showed that there was no significant association between educational status and vitamin D (p=0.640), and that the previous association had been due to education acting as a proxy for group status. On the adjusted education status scale, devised to account for the fewer years of education in the Bangladeshis, there was also no association with vitamin D (p=0.313).

There was no association between religion and mean vitamin D in the sedentees (this was not analysed in whites due to lack of data on religion, and all of the migrants were Muslim). The 17 non-Muslims had a similar mean 25(OH)D to the Muslims (51.3 vs 50.4nmol/L respectively, p=0.829) and were no more likely to be insufficient in vitamin D (p=0.535).

In addition, no linear association was found between length of time since migration and vitamin D (p=0.638).

Use of either cigarettes or betel nut with tobacco was not significantly associated with Vitamin D levels (p=0.426). Use of either calcium or vitamin supplements was also not associated with significantly higher Vitamin D (p=0.786). These results are shown in table S5.

Table S6: Associations between lifestyle factors, migration status and vitamin D

|  |  | **Mean 25(OH)D (nmol/L)** | **p-value** |
| --- | --- | --- | --- |
| **Smoking or use of tobacco with betel nut^a^** | Either  n=43 | 48.5 | 0.426 |
|  | None  n=98 | 45.7 |  |
| **Supplements: combined^b^** | Take any  n=46 | 46.6 | 0.786 |
|  | Take none  n=97 | 45.7 |  |
| **Supplements: calcium^c^** | Yes  n=16 | 48.4 | 0.632 |
|  | No  n=128 | 46.0 |  |
| **Supplements: other vitamins and minerals^d^** | Yes  n=43 | 46.7 | 0.769 |
|  | No  n=104 | 45.7 |  |

^a^ missing data for n=3 white British, n=2 migrants and n=2 sedentees

^b^ missing data for n=1 white British, n=3 migrants, n=1 sedentee

^c^ missing data for n=1 white British, n=3 migrants

^d^ missing data for n=1 sedentee
